# Characterization of a Nagina22 rice mutant for heat tolerance and mapping of yield traits

**DOI:** 10.1186/1939-8433-6-36

**Published:** 2013-12-02

**Authors:** Yugandhar Poli, Ramana Kumari Basava, Madhusmita Panigrahy, Vishnu Prasanth Vinukonda, Nageswara Rao Dokula, Sitapathi Rao Voleti, Subrahmanyam Desiraju, Sarla Neelamraju

**Affiliations:** Directorate of Rice Research, Rajendranagar, Hyderabad, 500030 India; Rai Technology University, Bangalore, India

**Keywords:** Heat stress, Nagina22, Mutant, Spikelet fertility, SMA

## Abstract

**Background:**

Heat is one of the major factors that considerably limit rice production. Nagina 22 (N22) is a deep-rooted, drought and heat tolerant aus rice cultivar. This study reports the characterization of a previously isolated dark green leaf mutant N22-H-*dgl219 (NH219)* which showed reduced accumulation of reactive oxygen species in leaf under 40°C heat conditions.

The mutant was characterized for several traits in field under ambient (38°C) and heat stress (44°C) conditions by raising temperature artificially from flowering stage till maturity by covering plants with polythene sheets during dry season 2011. Yield traits were mapped in 70 F_2_ segregants of IR64 × *NH219* and 36 F_2_ segregants of its reciprocal cross.

**Results:**

Leaf proteome analysis using two-dimensional gel electrophoresis from N22 and *NH219* showed distinct constitutive expression of ribulose bisphosphate carboxylase large chain precursor (EC 4.1.1.39) in *NH219* under ambient growth condition. Heat stress resulted in reduction of all 11 traits except plant height in both N22 and *NH219*. The extent of reduction was more in N22 than in *NH219*. Both pollen viability and spikelet fertility were not reduced significantly in N22 and *NH219* but reduced by 20% in IR64.

**Conclusion:**

*NH219* is more tolerant to heat stress than wild type N22 as its percent yield reduction is lesser than N22. Single marker analysis showed significant association of RM1089 with number of tillers and yield per plant, RM423 with leaf senescence, RM584 with leaf width and RM229 with yield per plant.

**Electronic supplementary material:**

The online version of this article (doi:10.1186/1939-8433-6-36) contains supplementary material, which is available to authorized users.

## Background

High temperature is often one of the most limiting factors affecting plant growth and crop yield in tropical and sub tropical areas. There is a risk that increased global temperature will change the optimum sites and conditions for crop production and thus affect agriculture. Temperature is estimated to increase by 2-4°C by the end of 21^st^ century as per scenario of Intergovernmental Panel on Climate Change (IPCC) and it may be due to emission of green house gases (Smith and Olesen 2010) and by human activities and natural factors or by both Eitzinger et al. (2010). Rice is the most important staple food crop grown in the areas where temperature is optimum for rice production and heat stress may limit sustainable rice production in these areas Tian et al. (2009). The effect of high temperature on rice yield depends on several complex factors individually and in combination including genotype, the growth stage at which it encounters heat stress, duration of stress, time of the day/night, the prevalent conditions of water vapour deficit, wind velocity, radiation, ambient recovery conditions (Morita et al. 2005 Prasad et al. 2006 Jagadish et al. [Bibr CR9][Bibr CR10] Ye et al. 2011 and Zhou et al. 2012). However, flowering stage is the most heat sensitive stage as heat stress during this stage results in loss of yield due to low pollen fertility and low seed set. QTL have been mapped for heat tolerance in different rice populations at booting (Zhao et al. 2006) and flowering (Ye et al. 2011 Jagadish et al. 2010a Zhang et al. 2008,2009 and Xiao et al. 2011). QTLs for heat tolerance are reported on all chromosomes except 6 and 7 (Cao et al. 2003 Chen et al. 2008 Zhang et al. 2008,2009 Jagadish et al. 2010a Xiao et al. 2011).

Mutants are valuable resources for genetic variations in crop improvement. Historically the use of mutagenesis in breeding has involved forward genetic screens and the selection of individual mutants with improved traits and their incorporation into breeding programmes. The novel genetic variations obtained from either spontaneous or induced mutants using physical or chemical mutagens can be exploited in crop genetics and their application in functional genomics and molecular breeding (Krishnan et al. 2009 Jiang and Ramachandran 2010). Analysis of genetic mutations is one of the most effective techniques for investigating gene function. Genes controlling developmental and metabolic processes have been discovered in plants by mutational analysis (Miroslaw and Iwona 2003). Mapping a novel mutation to a well defined chromosomal region is an important step in genetic analysis. The International Rice Functional Genomics Consortium announced the public availability of more than 200,000 rice mutant lines, which represent mutations in about half of the known functional genes mapped for rice to date (Krishnan et al. 2009). Though Nagina 22 (N22) is deep rooted, drought and heat tolerant aus rice variety (Jagadish et al. 2010b), there are very few genetic studies using N22 mutants. Characterization of Ethyl Methane Sulphonate (EMS) induced mutants of N22 for water stress and heat tolerance was reported by Panigrahy et al. (2011). A dark green leaf mutant, N22-H-*dgl219 (NH219)* was isolated under prolonged drought. During dark-induced senescence, *NH219* maintained higher chlorophyll and carotenoid content and photochemical efficiency of photosystem II in comparison with N22. Detached leaves of *NH219* accumulated less reactive oxygen species (H_2_O_2_ and superoxide radicals) and maintained higher chlorophyll content than N22 after 40°C heat treatment for 3 days. The present study reports further characterization of *NH219* for heat tolerance under field conditions in comparison with its wild type N22. The mutant *NH219* was crossed with another moderately heat tolerant variety IR64 (Khush and Virk 2005 Jagadish et al. 2010b) to map the mutation causing the drought and heat tolerant phenotype in the mutant and heat tolerance associate traits. The results of initial mapping of mutations in *NH219* using F_2_ segregants with extreme phenotype for eight traits derived from both IR64 × *NH219* and its reciprocal cross are also reported.

## Results

### Characterization of N22, *NH219* and IR64 for heat tolerance

Morphological and physiological traits (plant height, tiller number, number of panicles, panicle length, yield/plant, pollen viability, spikelet fertility, chlorophyll a/b ratio (chl a/b), relative water content (RWC), electron transport rate (ETR) and Fv/Fm were studied in N22 and *NH219* in field in 2 sets, one in ambient conditions and the other in heat stress conditions (Table 1). All the observed trait values in ambient conditions were higher in mutant compared to N22 and IR64 except marginal difference in Chl a/b ratio and ETR. Heat stress resulted in reduction of all observed traits for all three genotypes except increase in plant height. However, the extent of reduction was higher in N22 and IR64 than in *NH219*. Under heat stress, the increase in plant height was more in *NH219* (12.82%) than in N22 (4.59%). Fv/Fm that indicates photochemical efficiency of PSII reduced in N22 and IR64 by 5.8% and 12.02% respectively but in *NH219*, no reduction was noticed. Significant reduction in yield per plant was observed under heat stress conditions in case of both N22 (33%) and *NH219* (23%).

**Table 1:** **Performance of N22,** ***NH219*** **and IR64 under ambient and heat stress conditions**

Treatment		Ambient Condition (38°C)				Heat stress condition (44°C)				% Reduction in trait value
Trait Name	Genotype	Mean	SD	Min	Max	Mean	SD	Min	Max	
Plant height (cm)	N22	82.80	7.10	72.00	97.00	86.60	3.78	80.00	91.00	−4.59
	*NH219*	83.85	7.51	71.00	100.00	94.60	4.77	84.00	100.00	−12.82**
No. of tillers	N22	13.05	3.41	7.00	20.00	9.80	2.94	6.00	15.00	24.90**
	*NH219*	15.55	4.31	10.00	26.00	12.30	4.42	7.00	22.00	20.90
No. of panicles	N22	12.65	3.44	7.00	19.00	7.30	1.57	6.00	11.00	42.29**
	*NH219*	15.15	4.16	10.00	26.00	10.40	3.34	7.00	18.00	31.35**
Panicle length (cm)	N22	17.10	3.26	12.00	24.00	13.85	3.02	10.00	18.00	19.01**
	*NH219*	18.75	2.00	15.00	22.00	16.80	2.25	13.00	20.00	10.40*
Yield/plant (g)	N22	1.14	0.04	1.08	1.20	0.76	0.07	0.65	0.85	33.24**
	*NH219*	1.45	0.02	1.41	1.48	1.11	0.07	1.03	1.20	23.35**
Pollen viability (%)	N22	78.95	4.72	76.08	84.40	74.6	6.65	68.70	81.80	5.51
	*NH219*	91.27	2.70	88.17	93.13	88.94	3.27	86.48	92.65	2.55
Spikelet fertility (%)	IR64	91.22	0.20	91.00	91.37	72.43	1.81	84.09	87.50	20.60*
	N22	95.08	2.50	92.31	98.53	90.08	5.13	81.00	93.22	5.26
	*NH219*	96.49	2.01	93.55	98.33	95.95	3.13	91.21	100.00	0.55
	IR64	97.57	1.28	95.65	99.04	73.20	2.54	69.23	75.53	24.98**
Chl a/b	N22	4.50	0.86	3.68	5.26	2.91	1.10	1.66	4.02	35.36
	*NH219*	4.02	0.79	3.18	4.92	3.11	0.62	2.57	3.82	22.60
	IR64	3.83	0.66	3.22	4.50	2.98	2.12	2.67	7.25	22.11
RWC	N22	92.90	1.84	87.88	94.62	90.77	7.68	75.39	99.54	2.29
	*NH219*	92.43	3.60	86.88	98.78	91.41	5.41	83.20	98.44	1.10
	IR64	91.19	5.83	80.95	98.73	90.3	4.04	87.03	99.51	0.98
ETR	N22	28.43	6.29	21.70	38.80	21.85	10.22	6.70	35.50	23.13**
	*NH219*	25.35	2.24	23.50	29.00	21.4	2.31	18.90	25.30	15.58
	IR64	28.53	1.18	26.80	29.80	23.85	4.67	15.60	27.00	16.39*
Fv/Fm	N22	0.75	0.02	0.73	0.78	0.71	0.06	0.61	0.76	5.87
	*NH219*	0.78	0.01	0.77	0.81	0.79	0.01	0.78	0.82	−0.89
	IR64	0.78	0.01	0.77	0.79	0.69	0.01	0.77	0.79	12.02**

*Chl* Chlorophyll, *RWC* Relative water content, *ETR* Electron transport rate.

**Significant at 0.01 level of probability; *Significant at 0.05 level of probability.

The differences between IR64 and *NH219* were not significant under normal ambient temperature for all observed traits but the results were significant under heat stress conditions for pollen viability, spikelet fertility and Fv/Fm ratio. Pollen viability and spikelet fertility were high in both IR64 and *NH219* under normal ambient temperature. However, in heat stress treatment, pollen viability and spikelet fertility in IR64 were significantly lower than in *NH219* and N22.

### Two-dimensional gel electrophoresis

**Figure 1:**
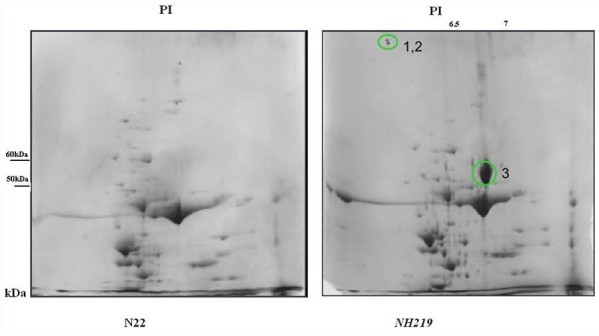
**2D gel electrophoresis picture of leaf proteins of N22 and** ***NH219*** **.** The conspicuous protein spot (spot 3) found between 50 kDa and 60 kDa and pI of 6.2 was identified as the Rubisco large chain precursor (EC 4.1.1.39).

Proteomic analysis was performed using 2-D gel electrophoresis of leaf samples of N22 and *NH219* to determine if they were constitutively different under ambient normal growth condition. In all, 54 spots showed differential expression above 2 fold when the 2 genotypes were compared. The analysis revealed the presence of a conspicuously large spot (spot 3) in *NH219* which was absent in N22. The PI value of this protein was 6.22 and nominal mass (Mr) was 53418. It matched to the score of 572 RBL_ORYSA (p < 0.05) and with sequence coverage of 55% with Ribulose bisphosphate carboxylase large chain precursor (EC 4.1.1.39) (P12089) (Figure 1). The other spots detected in the gel were not considered as their score was less than 39 and not significant (p > 0.05).

### Single marker analysis in F_2_ mapping population

The 70 F_2_ progeny of IR64 × *NH219* cross showed normal distribution in all measured agronomic traits except for number of tillers and leaf thickness which were skewed to the lower side of their mean values (Table 2). Similarly, in the 36 F_2_ population of *NH219* × IR64 cross the distribution of number of tillers and yield per plant was skewed to left side of the mean and more progeny showed lower number of tillers and low yield per plant. The other traits were normally distributed.

**Table 2:** **Performance of F**_**2**_ **population of IR64 ×** ***NH219*** **and its reciprocal cross**

F_2_of cross	IR64 × ***NH219***							***NH219*** × IR64						
Trait/Source	N	Mean	SD	Min	Max	Skew	Kurtosis	N	Mean	SD	Min	Max	Skew	Kurtosis
Plant height (cm)	70	75.01	17.00	42	123	0.39	−0.04	36	78.37	14.5	59	117	0.80	0.14
No. of tillers	70	4.64	2.87	1	16	1.67	3.53	36	5.09	2.47	2	12	1.36	1.94
Leaf width (cm)	70	1.26	0.30	0.50	1.80	−0.25	−0.23	36	1.22	0.21	0.80	1.70	0.13	−0.31
SPAD value	70	33.82	9.91	18.5	58.2	0.87	0.17	36	37.81	14.9	18.5	59.6	−0.11	−1.88
Leaf temperature (°C)	70	24.27	2.61	22.2	30.3	0.73	0.00	36	24.51	2.83	20.2	30.3	0.57	−0.53
6 day senescence	70	18.63	11.8	1.50	41.3	0.21	−1.10	36	20.85	8.53	2.1	37.4	−0.60	−0.03
Leaf thickness (mm)	70	0.46	0.22	0.19	1.26	1.36	2.62	36	0.44	0.19	0.19	0.92	0.94	0.06
Yield/plant (g)	70	2.52	1.97	0.00	8.00	0.88	0.34	36	2.59	2.34	0.3	10.4	1.57	2.54

**Figure 2:**
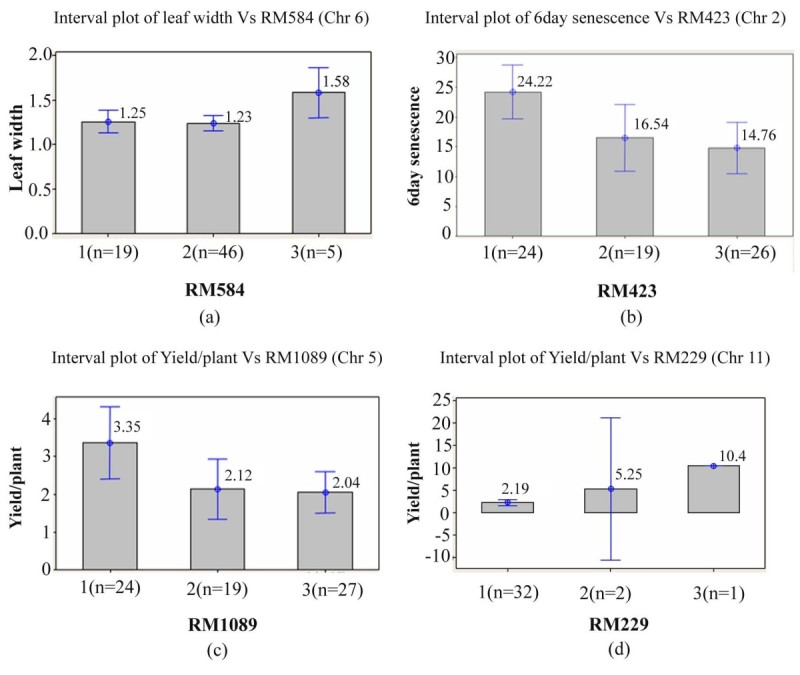
**Interval plot of significant trait vs marker association in F**_**2**_**population using single marker analysis. (a)** Leaf width vs RM584 **(b)** 6 day senescence vs RM423 **(c)** Yield per plant vs RM1089 **(d)** Yield per plant vs RM229. The data above the bar is mean value of trait for each genotype. The interval bar shows 95% confidence interval of mean. Genotype 1 = *NH219*, 2 = IR64, 3 = Heterozygote.

Nine markers showing polymorphism between IR64 and *NH219* were initially used for genotyping all the F_2_ plants to know if they segregated in a 1:1 ratio. Two markers RM584 (chromosome 6) and RM324 (chromosome 2) showed 31% and 26% *NH219* alleles respectively and a third marker RM229 (chromosome 11) showed 93% *NH219* alleles. Based on SMA (one-way Anova) of both F_2_ populations, four markers out of 9 showed significant differences among IR64 genotype, *NH219* genotype and heterozygotes (Table 3). The interval plots of these markers with their significant traits at 95% confidence interval level are shown in Figure 2.

**Table 3:** **Significant results of single marker analysis of F**_**2**_ **population**

Trait	Marker	Chromosome	Position (bp)	F	P	SD	N
Leaf width	RM 584	6	3416595-3416745	3.47	0.04	0.28	70
6 day senescence	RM 423	2	3836613-3836866	5.06	0.01	10.96	70
Number of tillers	RM 1089	5	5356155- 5356374	3.61	0.03	2.77	70
Yield per plant	RM 1089	5	5356155- 5356374	3.97	0.02	1.79	70
	RM 229	11	21019810--21019921	11.96	0	1.83	36

Map position of the marker was based on Nipponbare genomic sequence (RAP-DB, http://rapdb.dna.affrc.go.jp/).

Considering the populations separately, the SMA results of F_2_ population of cross IR64 × *NH219* showed that the mean leaf width of each genotypic class at locus RM584 was as heterozygote type (1.58) > *NH219* genotype (1.25) > IR64 genotype (1.23). The alleles contributing for leaf width may come from *NH219*. At marker RM1089, the mean number of tillers was significant between IR64 genotype (6.05) and *NH219* (3.83) and the mean yield (per plant) was significant between *NH219* (3.35) and heterozygote (2.04). These results indicate that the alleles for more number of tillers and high yield come from heat susceptible parent IR64 and heat tolerant *NH219* parent respectively at SSR locus RM1089. At marker RM423, the leaf senescence causing allele was contributed from N22 mutant parent as the mean value of this trait for *NH219* genotype (24.2) was considerably more than that of heterozygote (14.8).

In case of the reciprocal cross *NH219* × IR64, only one marker locus RM229 showed significant difference in yield per plant between genotypes with IR64 allele and those with *NH219* allele. The mean of yield at this marker locus for each genotype class was IR64 type (10.4) > heterozygote type (5.25) > *NH219* type (2.19). The yield increasing allele is thus derived from the susceptible parent IR64.

## Discussion

N22 is well known as drought and heat tolerant cultivar and used in breeding programmes for drought tolerance. (Jagadish et al. 2010b). In the present study, N22 and its EMS induced dark green leaf mutant, *NH219* were further characterized for morphological and physiological parameters under heat stress treatment in field and compared with N22. Changes in plant architecture play a significant role in adaptation to heat stress. Plants with panicles surrounded by many leaves can tolerate high temperature due to transpiration cooling effect of leaves which reduces the water evaporation from anthers and thereby increases anther dehiscence (Shah et al. 2011). Increase in plant height increases transpiration cooling effect and helps in avoidance from high temperature in mungbean and wheat (Kumar et al. 2011 Hasanuzzaman et al. 2013). A similar increase in plant height was observed in N22 and its mutant *NH219* under heat stress and the increase was more in the mutant.

Heat stress resulted in reduction of number of tillers, number of panicles and panicle length in both N22 and *NH219*. However, the extent of reduction was lesser in *NH219* showing its ability to withstand heat stress better. Pollen viability is an important trait as it is influenced by high temperature directly before it is shed and post anther dehiscence less than one hour exposure to temperature above optimum was sufficient to induce pollen sterility (Matsui et al. 1997 Jagadish et al. 2007). Zhou et al. (2012) reported large differences in floret fertility among rice genotypes subjected to heat stress. High temperature induced abnormal anther dehiscence leading to reduction in number of germinated pollen on stigma and resulting in spikelet sterility. N22 accessions showed significantly higher spikelet fertility under high temperature when compared with that of Moroberekan, a heat sensitive variety (Rang et al. 2011). Pollen viability and spikelet fertility were almost similar in N22, *NH219* and IR64 under ambient growth condition, however after heat stress it were maintained in the mutant compared to 5% reduction in N22 and 21% reduction in IR64. N22 is a drought and heat tolerant variety and the present experiments were carried out in summer season at DRR, Hyderabad, India where the peak summer temperature during April and May ranges from 35 to 42°C. The plants experienced this gradual increase in temperature from the vegetative stage itself and may have got acclimatized to high temperature. Hence, even though the plants were exposed to 5-8°C higher temperature inside the polythene tunnel, there was only 5% reduction in pollen viability and spikelet fertility. Since gametogenesis takes place much before emergence of the panicle, it is possible that this critical stage of pollen formation was not affected in N22 and *NH219* which flower a few days earlier than IR64. Further, the increase of 5-8°C inside the tunnel is in case of maximum temperature (around 12 pm−2 pm). However, in genotypes where anthesis, pollen dehiscence and germination occur in the early hours (before noon) it is possible they escape the adverse impact of heat stress. This could be one reason for low reduction in pollen viability and seed set. However, there was significant reduction in yield per plant in both N22 as well in *NH219* in heat stress conditions. Mohammed and Tarpley (2009) reported that rice plants grown under high night temperature showed 90% decrease in yield compared to plants grown under ambient temperature.

High temperature reduced chlorophyll content and the reduction was more in thermo-sensitive genotypes (Zhou et al. 2012). The current study revealed decrease in both total chlorophyll content and chlorophyll a/b ratio in case of N22, but an increase in chlorophyll content in *NH219* (Panigrahy et al. 2011) and lesser decrease in chlorophyll a/b ratio. Relative water content in leaf was reduced slightly in both genotypes which might be due to increased transpiration in heat stress. Wahid and Close (2007) reported that leaf water potential changed in high temperature conditions even though the soil water supply and relative humidity were normal. The PSII photochemical efficiency (Fv/Fm) was shown to reduce in rice seedlings in high temperature (Han et al., 2009). No significant reduction in Fv/Fm was observed in *NH219* due to heat stress indicating no damage to photosystem II complex or primary photochemical efficiency which was affected maximum in the susceptible line IR64 and least in the mutant *NH219*.

Ribulose bisphosphate carboxylase oxygenase (Rubisco) is a heat-labile protein in many plant species, limiting photosynthetic capacity during heat stress (Kurek et al. 2007). However, its increased abundance after heat treatment has been reported in heat tolerant rice species like O. *meridionalis* from Australia and its activity is associated with thermotolerance (Scafaro et al. 2010; Scafaro et al. 2012). Also transgenic rice over-expressing Rubisco activaseI showed greater photosynthetic activity (Wu et al. 2007). Regeneration of RuBP was altered in high temperature due to disruption of electron transport and inactivation of the oxygen evolving enzymes of PSII (Parry et al. 2013). The stable chlorophyll thylakoid complexes under water stress condition and reduced accumulation of reactive oxygen species under heat treatment has been reported in *NH219* (Panigrahy et al. 2011). The presence of a large amount of long chain precursor of Rubisco in *NH219* in normal ambient conditions and its absence in wild type N22 indicates that the precursor in N22 is either unstable or quickly used up to form large sub unit in N22 but is more stable or overproduced in *NH219* constitutively. Thus overall heat tolerance of *NH219* can be partly explained by the presence of a large pool of this precursor which may compensate for the heat stress induced deactivation of large sub unit of Rubisco. Further experiments on presence of the precursor in heat stress conditions can confirm this hypothesis. The role of large subunit precursor in heat stress tolerance has not been reported. Studying the turnover in N22 and *NH219* in heat stress conditions would help determine its role.

Ye et al. 2011 reported two major QTLs for heat tolerance on chromosome 1 and 4, explaining variations in spikelet fertility in BC_1_F_1_ and F_2_ populations derived from IR64 × N22 cross. Four single nucleotide polymorphisms were linked to heat tolerance based selective genotyping and single marker analysis. SMA analysis for mapping of yield related traits showed distinct association of RM229 (chromosome 11) with yield per plant at 0.05% probability level. This marker was previously reported to be associated with yield in BC_2_F_2_ population of Caiapo an upland *O. sativa* japonica variety from Brazil and *O. rufipogon* from Malaysia (Moncada et al. 2001). RM229 was also linked to three QTLs for root growth traits in rice. (Yue et al. 2006). RM229 is located on physical map at 18407911–18407976 bp and is positioned within the locus LOC_Os11g32030 (18407869–18410840) and is flanked by LOC_Os11g23020 on the left and LOC_Os11g32040 on the right. LOC_Os11g32030 encodes for the Sex determination protein Tassel seed-2 which is putatively expressed in rice. This gene belongs to short-chain dehydrogenase/reductase (SDR) family. It is a short chain alcohol dehyderogenase and is required for stage specific floral organ abortion (downloaded from GRAMENE, IRGSP and RAP-DB). Tassel branch of mutants in maize have been shown to affect yield under drought stress environments (Mulungani 2010).

Another locus RM423 was associated with leaf senescence, an important character for heat tolerance to retain chlorophyll content and thereby photosynthetic efficiency. However, wild type allele of this locus from *O. rufipogon* showed negative phenotypic effect on days to flowering and 1000-seed weight (Cho et al. 2003). RM584 on chromosome 6 was significantly associated with leaf width in present study. It was earlier reported to be associated with grain yield, spikelet fertility, days to flowering and 1000-seed weight (Moncada et al. 2001). It is interesting to note that RM584 is less than 100 base pairs away from RM225 which was reported to be associated with pollen fertility measured using the same staining procedure as we used (Xiao et al. 2011). RM1089 on chromosome 5 was significantly associated with number of tillers and yield/plant. Cho et al. 2012 reported RM1089 flanked a QTL for culm length and days to flowering. Further mapping in this mutant can provide more insights regarding the causal mutation for the mutant phenotype.

## Conclusion

We conclude that the EMS induced mutant *NH219* can tolerate heat stress more when compared with its wild type N22. *NH219* showed lesser reduction in yield/plant and related traits compared to N22. Ribulose bisphosphate carboxylase large chain precursor (EC 4.1.1.39) was present in *NH219* leaves and absent in N22 under ambient growth conditions. Both pollen viability and spikelet fertility were significantly reduced in IR64 but not in N22 and *NH219*. Marker RM1089 was associated with number of tillers and yield per plant, RM423 with leaf senescence, RM584 with leaf width and RM229 with yield per plant, based on single marker analysis of F_2_ mapping population from the cross between IR64 and *NH219*. Dense genotyping of mapping population can help to map traits related to heat tolerance.

## Methods

### Characterization of N22, IR64 and *NH219* for heat tolerance in field conditions

**Figure 3:**
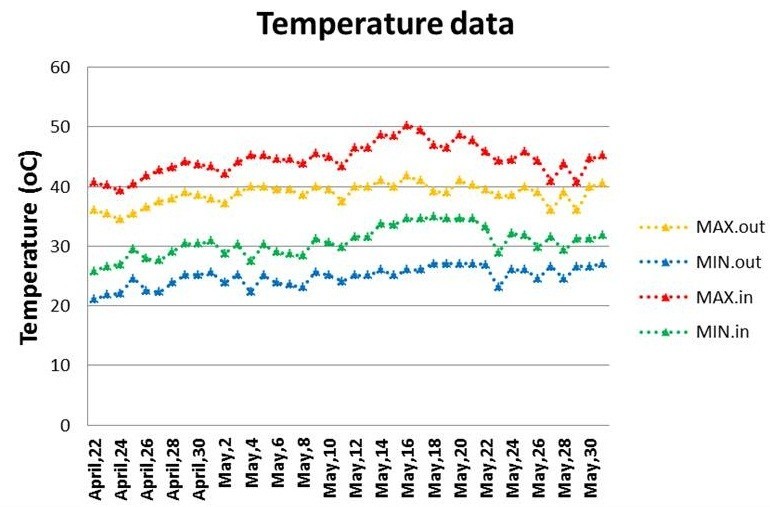
**Maximum and minimum temperature (°C) recorded 30** ^**th**^ **during the period of experiment (22** ^**nd**^ **April to May, 2011) in field under ambient conditions (out) and inside polythene tunnel (in).**

Nagina22 is a well known drought and heat tolerant aus variety where as IR64 is improved cultivated variety but reported to be heat susceptible, Jagadish et al. (2010b). IR64 was used for developing a mapping population. *NH219* is an EMS mutant of N22 (N) which was isolated in Hyderabad (H) as a dark green leaf mutant (NH_- dgl – 219 = *NH219* for short) under prolonged drought in M_5_ generation (Panigrahy et al. 2011). Heat stress experiments were conducted using 3 genotypes (N22, *NH219* and IR64) in DRR field (latitude and longitude: 17° 22′31″ N and 78° 28′27″E) under two environments viz., normal summer ambient temperature and heat stress conditions created artificially in the field during - summer season, 2011. For this study, plants were grown in 6 replications of 2 lines of each genotype and 22 plants per line with spacing of 20 cm × 20 cm. When the plants attained booting stage (22nd April), plants in 3 replications were covered with polythene sheet of 1 mm thickness (8-10% radiation decreased) to provide heat stress till maturity (30th May) as shown in Additional file 1. The day-night temperature, relative humidity (RH) and light intensity inside and outside polythene sheets were recorded during this period using thermohygrometer and photometer (N.S. Dimple Thermometers, Delhi, India). The ambient temperature in normal conditions was recorded as ≤ 41.8°C, ≥ 34.5°C, and average 38.7°C during day and ~24.9°C night. The RH under ambient normal conditions was ≤ 87%, ≥ 30%, average 60.1% and light intensity was 2.74 kW/m^2^. The temperature inside polythene sheet cover was ≤ 50.3°C, ≥ 38.3°C, average 44.6°C during day and ~30.7°C night. The details of temperature recorded during the experiment are shown in Figure 3. The RH was 5-8% higher inside the polythene tunnel than ambient conditions. At physiological maturity, the following morphological traits were studied in case of N22 and *NH219*, viz., plant height (length of the tallest tiller upto tip of panicle in cm), number of tillers, number of panicles (number of panicles with seeds exceeding 15%), length of panicle (length from neck to last spikelet of main panicle in cm), and yield/plant (mean weight of filled seeds from 22 plants). At flowering stage, the following physiological characters were studied for all three genotypes (N22, *NH219* and IR64).

Pollen viability: Pollen were harvested from 3 plants at random just before dehiscence and were stained on microscopic slide (200–300 grains counted per microscopic view and 3 views per slide) using 1% aqueous iodine-potassium iodide (I_2_KI) solution and analysed at 10× magnification using NIKON Alpha Shot-2 YS2H-E1112439 microscope. Complete dark staining of pollen with (I_2_KI) solution was considered as viable.

Spikelet fertility: Both partially and fully filled spikelets were considered as filled spikelets. Spikelet fertility was calculated as the ratio of filled spikelets to total number of spikelets.

Chlorophyll a/b ratio: Pigments were extracted from mature leaf tissue by grinding to a fine powder, homogenized in 80% (v/v) acetone, incubated at −20°C for 1 hour and centrifuged. The absorbance of the supernatant was measured at 645 and 663 nm using UV–VIS spectrophotometer Lambda 35 (Perkin Elmer, MA, USA). Chlorophyll a and Chlorophyll b concentration was calculated as:

$$\mathrm{Chl}\;a=12.25\;A_{663}-2.79\;A_{645}\;\mu g/\mathrm{ml}$$

$$\mathrm{Chl}\;b=21.5\;A_{645}-5.1\;A_{663}\;\mu g/\mathrm{ml}$$

Relative water content (RWC): Fresh leaf weight, turgid leaf weight (after soaking leaf in water for 16 hours) and dry leaf weight (after drying the leaf at 70°C for 72 hours) were measured. RWC was calculated using the formula;

$$\begin{array}{l} \mathrm{RWC}\;\left(\%\right)=\left(\mathrm{Fresh}\;\mathrm{weight}–\mathrm{Dry}\;\mathrm{weight}\right)\\ \;\times 100/\left(\mathrm{Turgid}\;\mathrm{weight}–\mathrm{Dry}\;\mathrm{weight}\right) \end{array}$$

Electron transport rate (ETR), and the ratio of variable fluorescence to maximum fluorescence (Fv/Fm) for leaf samples were measured using PAM-210 fluorescence meter (Walz, Effeltrich, Germany).

### Two dimensional gel electrophoresis

Two dimensional (2D) gel electrophoresis was carried out in three replicates with leaf samples of N22 and *NH219*, grown in field under normal ambient temperature condition during wet season. The leaf samples were outsourced for carrying out 2D gel electrophoresis, identification and analysis of differentially expressed spots by MS/MS to Vimta labs, Hyderabad, India.

### Phenotyping of F_2_ population

250 plants from F_2_ populations of IR64 × *NH219* and the reciprocal cross were grown singly in pots in net house under normal temperature and sunlight during wet season for phenotyping. Based on phenotype data, 70 and 36 F_2_ plants were selected from IR64 × *NH219* and the reciprocal cross respectively by including the extreme phenotype plants for each of the 8 traits. Observations were taken for eight traits viz., plant height, number of tillers, leaf width, leaf thickness (thickness of 3^rd^ leaf measured using Beta gauge Model 06-664-16. S/N: 101401673), SPAD value (SPAD 502 plus, Konika Minolta) on standing crop, six day senescence of leaf (by measuring SPAD value of leaf on 6th day after detaching it from plant and keeping it in long test tube with 30 ml water in dark at normal temperature) and temperature of leaf (using infrared thermometer, Fischer Scientific) at the maximum vegetative stage and yield per plant at maturity stage.

### Genotyping of F_2_ population and analysis

Genomic DNA of N22, IR64, *NH219* and 70 F_2_ plants of IR64 × *NH219* cross and 36 F_2_ plants of *NH219* × IR64 cross was isolated from leaves using Cetyl Trimethyl Ammonium Bromide extraction buffer. Genotyping was done for 70 samples of first cross using six SSR markers and 36 samples of second cross using three SSR markers. The markers were selected based on polymorphism between IR64 and N22 and also linked to QTLs of different agronomic traits. Single marker analysis (SMA) was done and the mean of each marker genotype was compared by one way Anova using MINITAB V14.0 (Minitab Inc., State College, PA, USA) to find out relation between each marker and each trait.

## Electronic supplementary material

Additional file 1: Field view of plants covered with polythene sheet to provide heat stress and uncovered ones serve as control set. (JPG 1 MB)

Below are the links to the authors’ original submitted files for images.

Authors’ original file for figure 1

Authors’ original file for figure 2

Authors’ original file for figure 3

## Abbreviations

NH219: N22-H-dgl219
Rubisco: Ribulose 1,5 biphosphate carboxylase oxygenase
RWC: Relative water content
ETR: Electron transport rate.
