# Supplementary figures and images for: Characterization of a Nagina22 rice mutant for heat tolerance and mapping of yield traits
Source: Rice (N Y). 2013 Dec 2;6:36. doi: 10.1186/1939-8433-6-36 (PMC4883711; doi:10.1186/1939-8433-6-36)

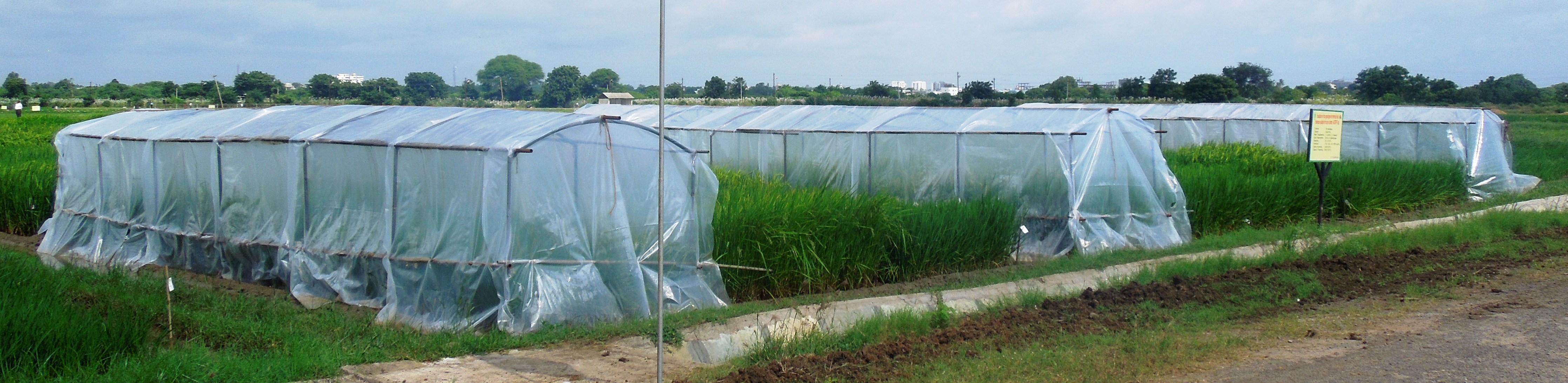

Supplement: Supplementary file 1 — Additional file 1: Field view of plants covered with polythene sheet to provide heat stress and uncovered ones serve as control set. (JPG 1 MB) [file 12284_2013_69_MOESM1_ESM.JPG]

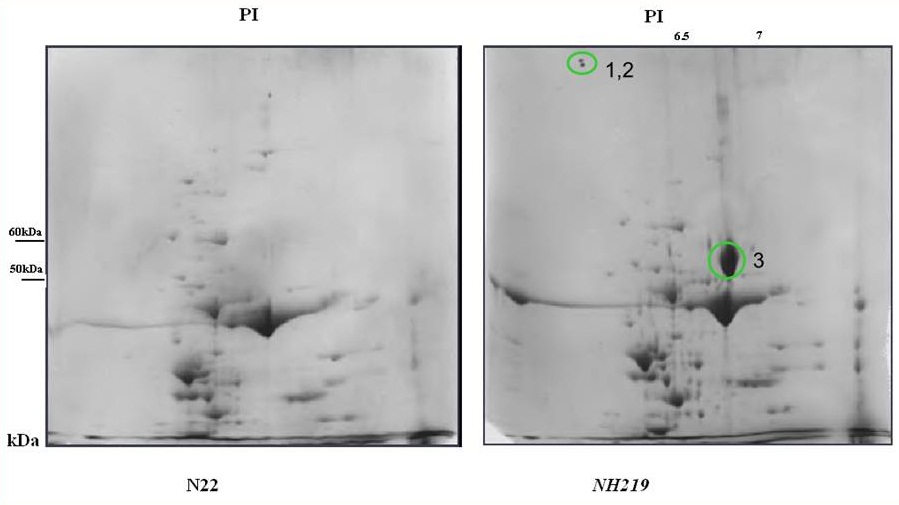

Supplement: Supplementary file 2 — Authors’ original file for figure 1 [file 12284_2013_69_MOESM2_ESM.jpg]

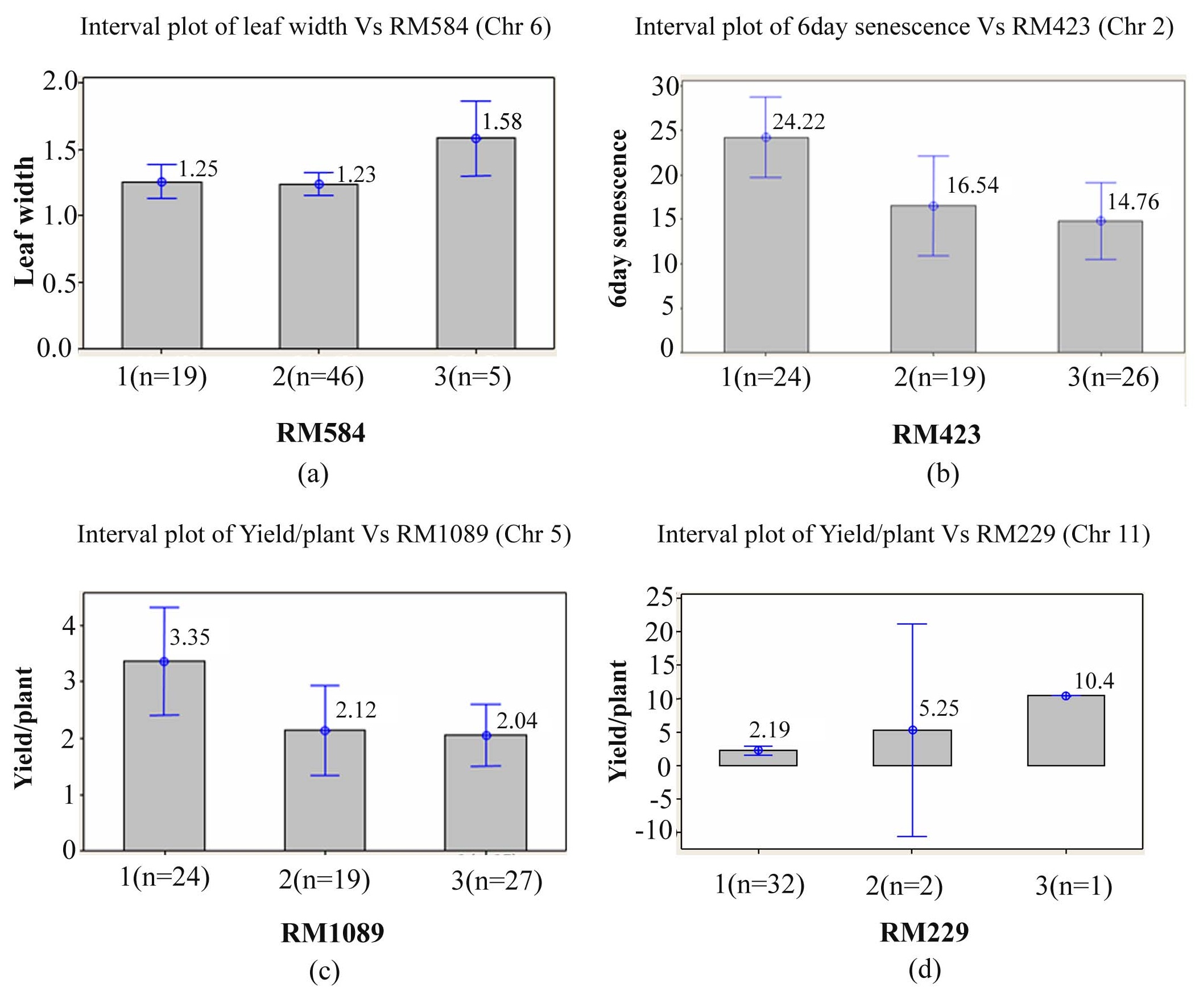

Supplement: Supplementary file 3 — Authors’ original file for figure 2 [file 12284_2013_69_MOESM3_ESM.jpg]

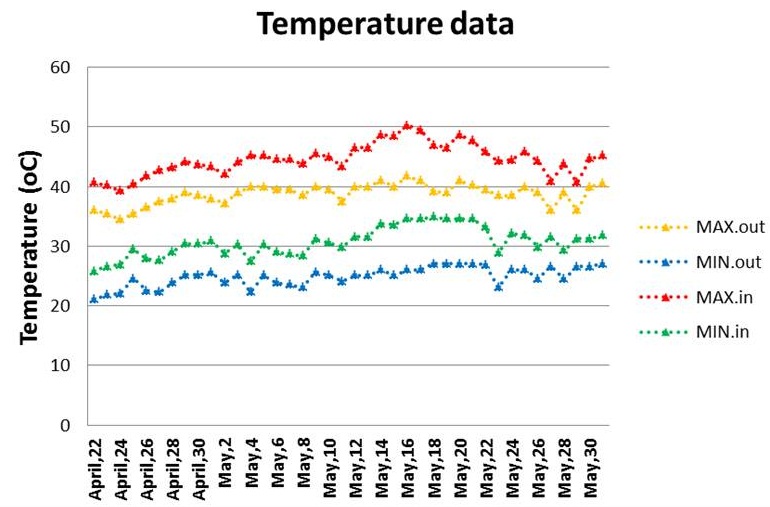

Supplement: Supplementary file 4 — Authors’ original file for figure 3 [file 12284_2013_69_MOESM4_ESM.jpg]
